# Expression of Pyruvate Carboxylase mRNA Variants in Liver of Dairy Cattle at Calving

**DOI:** 10.1371/journal.pone.0001270

**Published:** 2007-12-05

**Authors:** Cansu Agca, Shawn S. Donkin

**Affiliations:** Department of Animal Sciences, Purdue University, West Lafayette, Indiana, United States of America; University of Florida, United States of America

## Abstract

**Background:**

Bovine liver expresses six pyruvate carboxylase (PC) transcript variants, bPC5′A, bPC5′B, bPC5′C, bPC5′D, bPC5′E, and bPC5′F, which only differ at the 5′ untranslated region (UTR) and contain a common coding region. The objective of this experiment was to determine the profile and abundance of PC transcripts in bovine liver at calving.

**Methodology/Principal Findings:**

A ribonuclease protection assay (RPA) protocol was developed to simplify analysis of these variants and investigate the changes in abundance of each 5′ UTR transcript relative to total PC mRNA. Liver biopsy samples collected from seven cows on +1 d relative to calving were analyzed by RPA to determine the profile in PC 5′ UTR variants. Results show that all six bovine PC 5′ UTR variants are detected at calving. Data indicate that bovine PC 5′ UTR variant A is the most abundant, variants C and E are least abundant and expression of variants B, D and F is intermediate at calving.

**Conclusions:**

This manuscript describes a simplified RPA method that quantifies the abundance of six PC transcripts by using two riboprobes. The lack of uniformity in the pattern of PC 5′ UTR variants at calving suggests an additional complexity for control of bovine PC mRNA expression at calving that may be the result of transcriptional controls, variation in mRNA processing, or a combination of these processes.

## Introduction

Pyruvate carboxylase plays an essential role in many metabolic pathways including gluconeogenesis, lipogenesis, amino acid metabolism and neurotransmitter synthesis. PC is a flux-generating enzyme for several metabolic pathways and its activity is controlled by allosteric activation by acetyl-CoA, transcriptional activation, and translation of the PC primary transcripts [1]. Bovine PC mRNA is upregulated at calving [2], [3], is increased with feed restriction [4], and is elevated in response to increased glucose demands caused by phlorizin administration [5]. Glucagon, glucocorticoids, thyroid hormone and insulin regulate PC transcription rate and activity [6], [7], [8], [9]. Pyruvate carboxylase 5′ UTR variants have been identified for rat, mouse, human and bovine [10], [11], [12]. The PC 5′ UTR variants in nonruminants are the result of transcription initiation from multiple promoters and alternative splicing [6]. Information on the profile and origin of variants in bovine liver is not yet available.

Bovine liver PC contains six 5′ UTR variants [12]. These 5′ UTR variants may originate from multiple promoters and alternative splicing of primary transcripts but this possibility has not yet been confirmed for bovine. Bovine PC 5′ UTR variants are comprised of a combination of four mRNA segments that uniquely combine to form bPC5′A, bPC5′B, bPC5′C, bPC5′D, bPC5′E and bPC5′F which range in size from 68 to 363 nucleotides [12]. The objective of this study was to determine the profile of bovine PC 5′ UTR variants expressed in liver of dairy cattle at calving.

## Results

**Figure 1:**
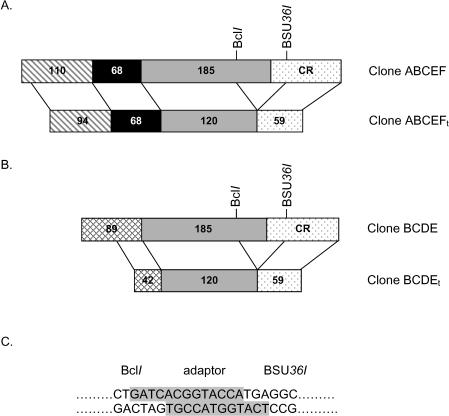
Construction of truncated RPA probes for bovine PC 5′ UTR variant analysis. Plasmid ABCEF containing all of the elements for bovine PC 5′ UTR was truncated by removing the 96 bp segment near the start of the PC coding sequence to form clone ABCEF_t_ (Panel A). Clone BCDE containing elements unique to bovine PC 5′UTRs B, C, D, and E was truncated by removing the 96 bp segment near the PC coding sequence to form clone BCDE_t_ (Panel B). The size of the UTR fragments of the parent and truncated clones is indicated within the shaded and patterned area. In both cases the segment removed included a portion of the 3′ end of the UTR and a portion of the 5′ end of the coding sequence. The coding region is indicated as CR. The adaptor for ligation of Bcl*I* and BSU*36I* digestions is shown in Panel C. The highlighted sequence represents the adaptor and plain text represents the sequence of the 5′ overhang end obtained from Bcl*I* and BSU*36I* digestion of plasmids ABCEF_t_ and BCDE_t_.

Two plasmids were constructed to generate truncated bovine PC plasmids to use as riboprobes for RPA analysis of bovine 5′UTR. The plasmid ABCEF was amplified from bPC5′C [12] but does not contain the first 13 bp of the 5′ UTR (Figure 1A). The plasmid ABCEF was named after the bovine PC 5′ UTR variants that it could bind. The plasmid BCDE was amplified from bPC5′E [12] but does not contain the first 47 bp of the 5′ UTR (Figure 1A). Similarly, the plasmid BCDE was named after the bovine PC 5′ UTR variants that it could bind. Both ABCEF and BCDE plasmids contain 90 bp of the coding region in addition to their respective 5′ UTRs. The truncated plasmids ABCEF and BCDE were generated by removing 65 bp upstream and 31 bp downstream of translation start site (Figure 1B). The truncated plasmids ABCEF_t_ and BCDE_t_ contained the respective truncated 5′ UTRs and an identical truncated coding region.

**Figure 2:**
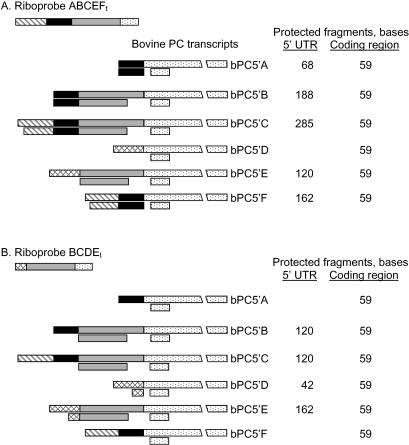
Hybridization pattern of riboprobes ABCEF_t_ and BCDE_t_ to bovine PC 5′ UTR variants. Panels A and B describe hybridization pattern of riboprobes ABCEF_t_ and BCDE_t_, respectively. The full sequence of each bovine 5′ UTR variant is represented by the connected series of filled and shaded boxes (that are described in Figure 1) to the left of the variant name. The full length riboprobes are indicated in the top left corner of each panel. Pairing of riboprobe segments with each 5′ UTR variant is indicated below the boxes representing each variant. Upon digestion with RNase H and T1 the sense and antisense paired strands of RNA remaining in solution are indicated for each UTR variant and coding sequence. The sizes of the protected fragments that originate from hybridization with each riboprobe are given on the right. Both probes protect an identical 59 nucleotide fragment of the coding sequence of bovine PC.

All bovine PC 5′ UTR variants were detectable by RPA analysis using riboprobes ABCEF_t_ and BCDE_t_. Because of the complexity of bovine PC 5′ UTR variants each probe protects several different fragments and each protected fragment represents either an intact 5′ UTR, a segment of one of the 5′ UTRs, or the protected fragment of the coding region (Figure 2). For example, the riboprobe ABCEF_t_ protects 59, 68, 120, 162, 188, 285 nucleotide fragments which represent the abundance of coding region, bPC5′A, bPC5′E, bPC5′F, bPC5′B and bPC5′C, respectively (Figure 2A). The riboprobe BCDE_t_ protects 42, 59, 120 and 162 nucleotide fragments which represent the abundance of bPC5′D, coding region, combination of bPC5′B and bPC5′C and bPC5′E, respectively (Figure 2B).

The protected fragments vary in length and nucleotide composition and intensity of the protected RNA fragment is directly proportional to the size and amount of ^32^P uracil residues in the protected fragment. Therefore, the intensity of each protected fragment was adjusted to account for these differences. The abundance of each 5′ UTR variant or coding region was determined by dividing the intensity by the number of uracil residues in each protected segment. Abundance of coding region for each sample was used to correct for differences between 2 probes and to determine the contribution of each mRNA variant to total PC mRNA. Previously we confirmed the presence of PC 5′ UTR variants by RPA using six riboprobes [12] where each 5′ UTR variant was detected using a probe unique for that particular variant. Analysis using the two riboprobes described here requires less sample and simplifies riboprobe synthesis and handling.

**Figure 3:**
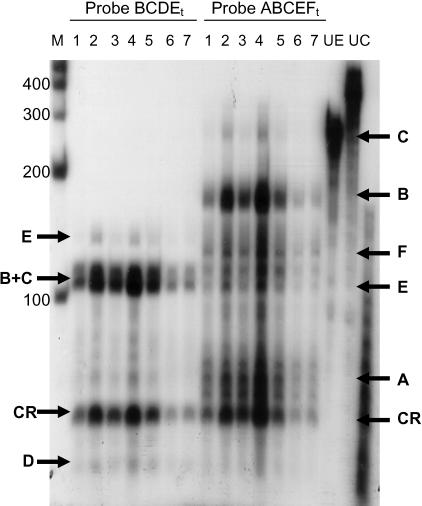
Autoradiogram of ribonuclease protection assay for bovine 5′ UTR variants. Total RNA samples were hybridized in solution with either riboprobe ABCEF_t_ or BCDE_t_ and digested with a mixture of RNase A and RNase T1. The protected fragments were separated by electrophoresis through a 5.5% polyacrylamide, 7 M urea gel. The protected fragments were visualized by exposing the dried polyacrylamide gel to Kodak X-Omat film and quantified using Kodak 1D image analysis software (Version 2.0.1). Fragments for transcripts bPC5′A, bPC5′B, bPC5′C, bPC5′D, bPC5′E, and bPC5′F are indicated by the letters A, B, C, D, E, and F respectively. The abbreviations CR, M, UE, and UC represent the coding region, molecular weight markers, undigested probe BCDE_t_, and undigested probe ABCEF_t_, respectively. Size of molecular weight markers are shown on the left of the gel.

**Figure 4:**
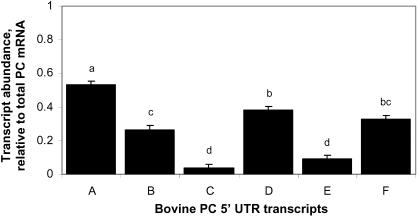
Contribution of PC 5′UTR variants to total PC mRNA on +1 d relative to calving. RNA samples were subjected to RPA analysis and the abundance of protected fragments was adjusted for the number of uracil residues within each fragment. Abundance of each variant was determined by dividing the uracil-adjusted values for each variant by the uracil-adjusted value of coding region generated from riboprobe ABCEF_t_ and riboprobe BCDE_t_. Means separation was by Duncan's Multiple Range Test and means that are statistically different (P<0.05) are indicated by superscripts (a, b, c, and d) above the bars that differ.

Figure 3A shows the autoradiogram of RPA analysis for RNA obtained from liver biopsy samples from seven cows on +1 day relative to calving. The Pearson correlation coefficient for band intensity corresponding to the coding region determined using probe ABCEF_t_ and probe BCDE_t_ was 95% (P<0.05). Due to the agreement between the data for probe ABCEF_t_ and probe BCDE_t_ for abundance of the coding region of PC mRNA the abundance for UTR segments relative to the coding sequence was combined across both probes. Analysis of the ratio of each PC variant to total PC mRNA indicates that variant bPC5′A contributes the most and variants bPC5′C and bPC5′E contribute the least to total PC mRNA (Figure 4). bPC5′A, bPC5′B, bPC5′C, bPC5′D, bPC5′E, bPC5′F contribute 53, 27, 4, 37, 9, 33±2% to total PC mRNA, respectively.

## Discussion

Pyruvate carboxylase is a rate limiting enzyme in gluconeogenesis. Gluconeogenesis is particularly important for dairy cows since they produce large amount of glucose (approximately 3 kg/day; [13]. Because the activity of PC positively correlates with PC mRNA abundance [2], [3], the profile of 5′UTR variants is important for determining the roles of each variant in PC control. In this paper we describe a simplified RPA method to determine relative abundance of each PC 5′UTR transcript. Determination of abundance of PC 5′ UTR transcripts presents a challenge since simpler methods such as real time RT-PCR would not be suitable due to the presence of common sequence within the 5′ UTRs and length of the UTR fragments. Bovine PC 5′UTR transcripts (with the exception of bPC5′D and bPC5′E) do not have unique sequences that would distinguish a single 5′UTR. Most primer pairs for real time RT-PCR would amplify at least two PC 5′ UTR transcripts and thus confound determination of level of a single variant. For example primer pairs that amplify bPC5′A, bPC5′B and bPC5′C would also amplify bPC5′F, bPC5′C and bPC5′A, respectively. Although RPA is a relatively labor intensive technique, it is the most reliable method to determine the abundance of each PC 5′UTR variant. In addition, use of two (riboprobes ABCEF_t_ and BCDE_t_) reduced number of samples three times compared to using individual riboprobes for each PC 5′UTR variant.

Rat and human PC mRNA have also multiple 5′ UTR variants that are transcribed from multiple proximal promoters of a single gene and alternatively spliced [14]. The biological significance of these 5′ UTR variants has been characterized for the rat. The unique PC transcripts exhibit different rates of protein translation and association with ribosomal subunits due to the inherent structures within their 5′-untranslated regions [1]. As a consequence, PC activity is controlled at the level of gene transcription, the profile of mRNA variants expressed, through allosteric regulation and through availability of biotin [1].

Control of protein expression as a consequence of mRNA 5′ UTR variants is also observed for growth hormone receptor [15], [16], acetyl Coenzyme A carboxylase [17] and neuropeptide Y receptor [18]. For these examples, variation within the 5′ UTR region of mRNA originates from either multiple promoters, alternative splicing of primary transcript or a combination of both processes. The presence of multiple promoters enables tissue specific expression [1] and differential rates of translation [18], [19]. In addition, 5′ UTR variants alter mRNA stability as observed for acetyl-CoA carboxylase [17]. Tissue specific expression has been determined for bovine PC 5′ UTR variants where all variants are present in gluconeogenic and lipogenic tissues, and other tissues contain only bPC5′B, bPC5′C, bPC5′D, and bPC5′E [12] although the effects of these UTR variants on translational capacity and RNA stability have not been fully characterized for bovine. Data on the profile of PC variants with physiological state and information on their capacities for PC enzyme synthesis should provide a more complete picture of the regulation of PC activity in response to physiological cues.

The biological significance of bovine PC 5′ UTR variants has not been fully determined but preliminary evidence suggests differences in translational efficiencies for these mRNA variants. The data presented here indicates greater abundance of bPC 5′A at calving. If differential translational capacity exists for bovine PC variants then the activity of the PC enzyme in liver at calving may be determined not only by the abundance of total PC mRNA but also by the profile of PC 5′UTR variants expressed.

### Conclusions

The presence of 5′ UTR variants may control protein synthesis by modulating translational efficiency and mRNA stability. Determining the abundance of 5′ UTR variants for specific physiological states may be important in assessing the cause and impact of changes in PC mRNA abundance. Data presented here indicates that all bovine PC 5′ UTR variants can be detected and quantified by RPA analysis using two related riboprobes, that all variants are expressed at calving, and variant bPC5′A is the most abundant and bPC5′E is the least abundant transcript.

## Methods

### Plasmid construction

Liver samples from Holstein cows were collected on +1 day relative to calving as part of a previously reported study [2] and used to synthesize the riboprobes necessary for PC 5′ UTR variant analysis. Total RNA was isolated using the phenol:chloroform:isoamyl alcohol extraction [20]. First strand cDNA was synthesized from total RNA using Superscript II reverse transcriptase (Invitrogen, CA). A gene specific primer which anneals between 180 and 203 bases downstream of translation start site (GGTAGGCTTCGTCAGCTTTCTGTC) was used in RT reaction and the product of this reaction was used in subsequent PCR reactions. Platinum Taq (Invitrogen, CA) was used for the PCR reactions and the conditions were: 3 minutes at 95°C initial denaturation followed by 35 cycles of 45 s at 94°C, 45 s at 58°C, 45 s at 72°C and 7 minutes at 72°C final extension. The forward primer for amplification of bPC5′C cDNA was AAGAATTCTCCCCTGAGCTGTAAGG which annealed between 350 and 331 bases upstream of translation start site [12]. The reverse primer was AAGGATCCGGACATTGGGAGAGGC and annealed between 73 and 90 bases downstream of translation start site [12]. The product of this reaction, clone ABCEF, was named for the transcripts that are quantified using this probe. The forward primer for amplification of bPC5′E cDNA was AAGAATTCGGAGATAGTGCCTGCC and annealed between 42 and 27 bases upstream of translation start site. The reverse primer was identical to the reverse primer for amplification of clone ABCEF. The product of this reaction, clone BCDE was named for the transcripts that are quantified using this probe. The forward primers contained Eco*RI* site and reverse primer contained Bam*HI* site to provide sticky ends for subsequent cloning to pGEM 3Z vector (Promega, Madison, WI) and these restriction enzyme sites are indicated by the underlined bases in the primers described in the preceding text.

The plasmids containing either clone ABCEF or clone BCDE were verified by sequencing using M13 forward and reverse primers using a Pharmacia ALFExpress DNA sequencer (Pharamcia Biotech Inc., Piscataway, NJ). The 5′ UTR sequence of clone ABCEF is based on bPC5′C [12] but lacks the first 13 bp of the 5′ UTR. The 5′ UTR sequence of clone BCDE is based on bPC5′E [12] without the first 47 bp of the 5′ UTR. Both clones (ABCEF and BCDE) contain 90 bp of the coding region.

The plasmids ABCEF and BCDE were then digested with BSU*36I* and Bcl*I* (Figures 1A and 1B) to remove a 96 bp fragment that includes 65 bp upstream and 31 bp downstream of translation start site. The incompatible ends from each digest were ligated using an adaptor sequence (Figure 1C) and the resulting truncated plasmids, ABCEF_t_ and BCDE_t_ contained the respective truncated 5′ UTRs and an identical truncated coding region.

### Ribonuclease protection assay

Total RNA samples from seven Holstein cows were used in RPA analysis. The samples were collected on +1 d relative to parturition as a part of previously reported study [2] and only samples from control animals were used in these analysis. Plasmids ABCEF_t_ and BCDE_t_ were linearized using Eco*RI* and ^32^P UTP-labeled antisense riboprobes were generated using SP6 RNA polymerase and the MAXIscript in vitro transcription kit (Ambion, Austin, TX). Riboprobes ABCEF_t_ and BCDE_t_ were hybridized overnight with separate 20 µg aliquots of total RNA according to the Ambion RPA III kit (Ambion, Austin, TX). Yeast RNA was included in the control reactions. The hybridization reactions were then incubated with a mixture of RNase A and T1. Using these conditions PC, mRNA variants were hybridized in solution with their corresponding ^32^P-labeled antisense strand and were protected from RNase A and T1 RNase digestion. The resulting digestion products were separated by electrophoresis through a 5.5% polyacrylamide, 7 M urea gel. The protected RNA fragments and control reactions were visualized by exposing the dried polyacrylamide gel to Kodak X-Omat film for 96 h. Intensities of the protected fragments were determined by Kodak 1D image analysis software (Version 2.0.1).

### Statistical analysis

Intensity of protected fragments was determined separately for ABCEF_t_ and BCDE_t_. Correlation of the intensity the coding region was determined between riboprobes using the Proc corr procedure of SAS. Data for the abundance of variants relative to the coding region was combined and compared in a single analysis. Differences in expression of transcripts on +1 day of lactation were determined by the GLM procedure of SAS (Release 8.2) and accounted for the variation associated with cow. Differences between variants were determined using the Duncan procedure.
